# Utility of Two Cancer Organization Websites for a Multiethnic, Public Hospital Oncology Population: Comparative Cross-Sectional Survey

**DOI:** 10.2196/jmir.7.3.e28

**Published:** 2005-07-01

**Authors:** Katherine D Nguyen, Belinda Hara, Rowan T Chlebowski

**Affiliations:** ^1^Los Angeles Biomedical Research InstituteHarbor UCLA Medical CenterTorrance, CAUSA

**Keywords:** Internet, Internet access, computers, cancer, cancer website, race, racial/ethnic minority

## Abstract

**Background:**

While information websites have been developed by major cancer organizations, their appropriateness for patients in multiethnic, multilingual public hospital settings has received limited attention.

**Objective:**

The objective of the study was to determine the utility of cancer information websites for a public hospital patient population.

**Methods:**

A 70-item questionnaire was developed to evaluate cancer information seeking behavior, Internet access and use, and content appropriateness of two cancer information websites: People Living with Cancer from the American Society of Clinical Oncology (ASCO) and Breast Cancer Info from the Susan Komen Breast Cancer Foundation (SKF). Interviews were conducted with consecutive consenting oncology patients seen in a public hospital oncology clinic.

**Results:**

Fifty-nine persons participated in the survey. The response rate was 80%. Participants were Caucasian (25%), African American (19%), Hispanic (42%), and Asian/Pacific Islander (11%). English was the primary language in 53% of participants, 56% had a high school education or less, and 74% had an annual income less than US $35000. With respect to computer and Internet use, 71% had computer access, and 44% searched for cancer information online, with more being interested in obtaining online information in the future (63%). Participants who had computer access were likely to be English speaking (*P* = .04). Those less likely to have previously used a computer tended to have a lower annual income (*P* = .02) or to be males aged 55 years or older (*P* < .05). When shown sample content from the two websites, almost all participants stated that it was “easy to understand” (ASCO 96%, SKF 96%) and had “easy to understand terms” (ASCO 94%, SKF 92%). Somewhat fewer respondents agreed that the websites provided “information they could use” (ASCO 88%, SKF 80%) or that they would return to these websites (ASCO 73%, SKF 68%). The majority planned to “discuss website information with their oncologists” (ASCO 82%, SKF 70%).

**Conclusions:**

Multiethnic, multilingual cancer patients at a public county hospital commonly had Internet access and found the content of two websites representative of major cancer organizations to be both understandable and useful.

## Introduction

The Internet has become an increasingly common source of medical information for patients with cancer, with 6% to 43% of this population now using this resource [1-3]. Major cancer organizations have created patient-centered websites to provide comprehensive information about specific cancers [4,5]. Use of the Internet to access cancer information has been correlated with younger age (age less than 60 years), higher income, higher education, and Caucasian race [1,6,7]. However, a recent review found little empirical research on Internet cancer information use among minority racial/ethnic groups or on the appropriateness of available websites for such populations [8]. We therefore developed a survey instrument in a multiethnic, multilingual public hospital population to explore Internet access and to determine the appropriateness of websites from two major cancer organizations: the People Living with Cancer website from American Society of Clinical Oncology (ASCO) and the Breast Cancer Info website from the Susan G Komen Breast Cancer Foundation (SKF).

## Methods

### Study Design and Eligibility

The survey was conducted between March 2003 and August 2003 at Harbor-UCLA Medical Center. A questionnaire was administered face-to-face by trained research interviewers to consecutive patients seen at the medical oncology clinic and the oncology infusion clinic areas. Eligibility was limited to oncology patients age 18 and older who were English or Spanish speaking and who were without major cognitive or physical impairment by physician assessment. Verbal consent was obtained from all participants, and information was collected without patient identifiers. The study protocol and consent process were approved by the institutional review board.

### Survey Instrument

The survey instrument was developed by initially conducting an extensive literature search to determine information seeking methods of cancer patients, including frequency of Internet use. Then, cancer care providers and a cancer social worker were interviewed to determine their perspectives and to inform the design of the questionnaire. Once a questionnaire was devised, a six-member expert panel including medical oncologists, cancer care nurses, and a cancer social worker evaluated content validity. Items deemed not relevant or incongruent were either deleted or revised.

The developed 70-item questionnaire has four sections. The first section (17 items) contains demographic information on age, sex, ethnicity, language, education, income, cancer type, and any current medical information seeking approaches. The second section (11 items) addresses baseline computer use, computer access, and attitudes toward computer use. The third section (22 items) examines the patient's unmet content needs, including satisfaction with the medical oncology clinic, medical information needs, any language barriers, and social support service needs. The fourth section (20 items) evaluates the usability and content of two websites. Participants were presented printouts of sample information from two major cancer websites, the ASCO People Living with Cancer website [4] and the SKF Breast Cancer Info website [5]. No prior computer or Internet experience was presumed, and participants were instructed to focus on information content as presented and not on the Internet or computer aspect. Literacy level was also not presumed as research associates read scripted information from the website to each participant. Content and usability were evaluated in terms of the participants' interest in the content, their ease in understanding the material and finding information, their assessment of the utility of the information, and their likelihood of discussing the information with medical providers in the future.

Spanish-speaking patients were known to account for a large proportion of the patient population; therefore, a Spanish questionnaire was developed. Spanish-speaking research interviewers were available to administer the Spanish-version questionnaire to patients identifying themselves as Spanish speaking. Questionnaires and interpreters were not available for other languages.

Categories of ethnicity/race were based on a self-report from five offered categories: American Indian/Alaskan Native, Asian/Pacific Islander, Black/African American, Hispanic, and White.

### Subjects

Of the 86 persons approached for participation, 12 (14%) were ineligible mainly due to absence of a cancer diagnosis. An additional 15 were eligible but declined participation. The main reasons were “not enough time” and “not interested.” Therefore, a total of 59 out of 74 eligible persons participated (80% response).

### Analysis

Microsoft Access 2000 was used to compute descriptive statistics. For each item, the proportion of persons endorsing each response category was calculated, and descriptive statistics were generated. Items assessed using a 5-point Likert scale (strongly agree to strongly disagree) were categorized as agree or not agree. Differences in subjects' evaluation of the two websites were compared using the McNemar test. Differences in responses to the information seeking and computer use section were compared according to demographic characteristics (gender, age < or ≥ 55 years, English or non-English primary language, annual income < or ≥ $35000) and were evaluated with the Fisher exact test. Similarly, subjects' responses to the website evaluation section were compared according to the same demographic characteristics and were evaluated with the Fisher exact test. Interactions between pairs of demographic characteristics were assessed by stratification and the Breslow-Day test. Level of statistical significance was set at .05, and no formal adjustment was made for multiple statistical tests.

## Results

### Characteristics of Respondents

In terms of demographics, most participants were female (66%). Participants included Hispanics (42%), Caucasians (25%), African Americans (19%), and Asian/Pacific Islanders (11%). The primary language was English in 53% of participants, 56% had a high school education or less, and 74% had an annual income less than $35000. The mean age was 52.1 years.

In terms of cancer type, 51% had breast cancer, 14% had lung cancer, and 10% had colorectal cancer. The mean time from cancer diagnosis was 2.5 years, with a range of 2 months to 15 years. Many respondents identified cancer as their only medical condition (51%). Other common medical conditions were hypertension (21%) and diabetes (14%).

### Information Seeking and Computer/Internet Access

Respondents identified additional sources of medical information, besides their physicians, including pamphlets (53%); friends, family, and other patients (48%); and the Internet (35%). Overall, 61% of participants had used a computer before, and 45% stated that they owned a computer. However, 71% stated they had computer access at locations such as home, work, the homes of friends and family, and the library. About 54% had used the Internet, and 44% had researched cancer online; a larger percentage (63%) stated interest in using the Internet as a cancer information source in the future, with particular interest in issues related to cancer treatment and emerging research. When asked if they could trust information from the Internet, 64% agreed online information could be trusted, while 27% were ambivalent (Table 1).

**Table 1:** Summary of information seeking and computer use

**Statement**		**Number(N = 59)**	**Percent**
Have used a computer before		36	61
Own a computer*		26	45
Have been online		32	54
Have access to computer		42	71
Where patient accesses computer†			
	Home	28	48
	Work	2	3
	Friend, family	15	25
	Library	7	12
	Other	3	5
	None	14	24
Have researched cancer online		26	44
Interested in cancer info online		37	63
Trust online information			
	Agree	38	64
	Disagree	5	8
	Neutral, do not know	16	27

^*^ N = 58

^†^ Respondents could include more than one answer; percents do not add to 100.

As Table 2 shows, fewer participants whose primary language was not English had computer access compared to those whose primary language was English (57% vs 84%; *P* = .04). This difference remained after stratifying by age, gender, and income. The combination of being male and older (at least 55 years) was significantly (*P* < .05) associated with being less likely to have used a computer before, to have been online, and to have an interest in cancer information online. Specifically, the percentages of females and males younger than 55 years, and females and males 55 years and older who had used computers were 68%, 67%, 80%, and 23%, respectively. The percentages who had been online (for the same categories of females and males as above) were 70%, 67%, 80%, and 8%. Similarly, the percentages who were interested in cancer information online were 83%, 83%, 77%, and 25%. There were no significant differences in computer use and Internet access when stratified by income level below $35000 versus $35000 or more. However, when using an income level of $20000, significant differences were observed. Fewer participants earning less than $20000 per year owned computers (29% vs 60%; *P* = .02) or had used computers (45% vs 77%; *P* = .02) compared to other participants.

**Table 2:** Comparison of sample items by language, gender, and income level

**Item**	**Non-English Primary Language (%)**	**English Primary Language (%)**	***P* value**
Have computer access	57	84	.04
Info on ASCO website more useful as pamphlet	90	55	.01
Info on SKF website more useful as pamphlet	86	45	.004
Info on ASCO website more useful as pamphlet in my language	81	10	< .0001
Info on SKF website more useful as pamphlet in my language	76	10	< .0001
	**Female (%)**	**Male (%)**	
Find info I need easily on ASCO site	89	50	.006
Would discuss ASCO site info with doctor	92	52	.006
SKF site has info I can use	89	54	.04
Plan to go back to SKF site	78	38	.01
	**Income< $35000 (%)**	**Income** ≥ **$35000** **(%)**	
Plan to discuss ASCO site with doctor	75	100	.04
Plan to discuss SKF site with doctor	64	86	.18
	**Income< $20000 (%)**	**Income** ≥ **$20000 (%)**	
Have ever used computer	45	77	.02
Own a computer	29	60	.02

### Evaluation of Websites

Participants' evaluation of the People Living with Cancer website and the Breast Cancer Info website is outlined in Table 3. As seen, when shown sample content from the two websites, the majority stated that it was “easy to understand” (ASCO 96%, SKF 96%) and had “easy to understand terms” (ASCO 94%, SKF 92%). Somewhat fewer agreed that the websites provided “information they could use” (ASCO 88%, SKF 80%), and that they would return to these websites (ASCO 73%, SKF 68%). A sizable proportion of the sample stated they would prefer that the information in the websites be presented in a printed pamphlet format (ASCO 69%, SKF 62%). The majority planned to “discuss website information with their oncologists” (ASCO 82%, SKF 70%). When asked if they would use this information to inform their medical decisions, most agreed (ASCO 69%, SKF 70%). There were no statistically significant differences between subjects' evaluation of the two websites.

**Table 3:** Evaluation of the American Society of Clinical Oncology and Susan G Komen Breast Cancer Foundation websites (n = 50)

	**ASCO Website**		**SKF Website**		***P* value**	
**Statement**	**Number Agreeing**	**Percent**	**Number Agreeing**	**Percent**
This website is easy to understand	49	96	48	96	.32
This use of medical terms and explanation is easy to understand	48	94	46	92	.32
I can find information I need easily	41	82	41	82	.32
This website has information I can use	45	88	40	80	.06
I plan to go back to this website	37	73	34	68	.32
The information on the website would be more useful to me as a printed pamphlet	35	69	31	62	.48
The information on the website would be more useful to me as a printed pamphlet in my language	19	37	19	38	.65
I would use this information to make medical decisions	35	69	35	70	.65
I plan to discuss information from this website with my doctor	41	82	35	70	.06

More women than men reported that on the ASCO website they could “find information I need easily” (89% vs 50%; *P* = .006), and that they would “discuss information from this website with my doctor” (92% vs 52%; *P* = .006). Proportionally more women than men stated that the SKF website “has information that I can use” (89% vs 54%; *P* = .04); that they “plan to go back to this website” (78% vs 38%; *P* = .01); and that “the information on the website would be more useful to me as a printed pamphlet” (73% vs 31%; *P* = .02). Significantly more participants whose primary language was not English reported that “the information on the website would be more useful to me as a printed pamphlet” (SKF: 86% vs 45%; *P* = .004; ASCO: 90% vs 55%; *P* = .01), and that “the information on the website would be more useful to me as a printed pamphlet in my language” (SKF: 76% vs 10%; *P* < .0001; ASCO: 81% vs 10%; *P* < .0001). There was a tendency for fewer participants with annual incomes less than $35000 to “plan to discuss information from this website with my doctor” (ASCO: 75% vs 100%; *P* = .04; SKF: 64% vs 86%; *P* = .18). There were no significant differences in evaluation of the websites when stratified according to age.

## Discussion

The results of our survey suggest that multiethnic, multilingual cancer patients at a public county hospital commonly have Internet access and find the content of two websites representative of major cancer organizations (the American Society of Clinical Oncology and the Susan G Komen Breast Cancer Foundation) to be both understandable and useful.

### Internet Use to Access Cancer Information

Medical information seeking on the Internet has been previously shown to statistically correlate with higher income, higher education, and non-minority race [1-3]. Our sample was predominantly non-Caucasian (75%), commonly had a high school education or less (56%), and had an annual income less than $35000 (74%). Nonetheless, 44% of this multiethnic, lower-income cancer population reported using the Internet to access medical information regarding their cancer, a result similar to that seen for more affluent cancer populations in which 6% to 43% of cancer patients have been reported to use the Internet for cancer-related information [6,9,10]. Our findings are also comparable to the frequency of Internet use for medical information reported in non-cancer populations. In two large surveys which focused on the general population (N = 3209) and on primary care patients (N = 512), using the Internet to access general medical information was reported for 31% and 54% of participants [9,11]. Such results suggest that the previously identified “digital divide”—less access to Internet information based on socioeconomic status [12]—may be decreasing as the Internet becomes increasingly available.

### Factors Influencing the “Digital Divide”

The “digital divide” in our population is influenced by language, age, gender, and income. Compared to participants whose primary language was English, those who reported another primary language were less likely to have computer access. This difference remained after stratifying by age, gender, and income. The combination of being male and 55 years or older was significantly associated with being less likely to have prior computer or Internet use or interest in cancer information online. There were no significant differences in computer use and Internet access by annual income level (above and below $35000) in this population. However, a difference was observed using a lower income level ($20000). Participants with an annual income below $20000 were significantly less likely to own a computer and to have used a computer compared to other participants.

### Utility of Website Information

In our study, when participants were shown sample pages from two major cancer organization websites, both sites received favorable overall reactions. Significantly, participants generally agreed that both were easy to understand and had information they could use. When asked what they would do with such information, the majority indicated that they would include website information in discussions with their physicians (82%) and in making medical decisions (69%). Even in our small sample, significant differences emerged between genders and reported primary languages in evaluation of the two websites. Significantly, more women than men stated that the ASCO website had information they could find easily and that the SKF website had information they could use. Not surprisingly, respondents' primary language influenced their preference for printed information versus website information. Compared to respondents whose primary language was English, persons who reported another primary language significantly preferred to have the website information as a pamphlet, particularly a pamphlet in their language.

Cancer patients seek information to regain a sense of control, learn about treatment, and inform their medical decisions [13-16]. The growth of the Internet has prompted concerns regarding the reliability of online medical information and the absence of a system to help patients navigate the vast numbers of websites or appraise their quality [10,17]. In fact, patients typically start their Internet search with a search engine and visit the first few sites listed [18]. Many major cancer organizations provide websites with comprehensive, current information that may be useful as reliable sources of patient education; however, patients may have difficulty finding such websites. To add another layer of complexity, little is known regarding how disadvantaged groups find reliable sites. Thus, new strategies are needed in order to help all cancer patients find reliable cancer information online.

### Strengths and Limitations of the Study

Strengths of this study include collection of detailed questionnaire information through interviews conducted in English and Spanish and evaluation of website content using a procedure independent of computer and language skills (a visual presentation of materials with spoken explanation). Study limitations include the modest sample size and findings based on self-report. The study targeted a specific multiethnic, multilingual, predominantly lower-income cancer population at one public hospital, and findings, therefore, cannot be generalized to other populations. Our study did not assess behavior after exposure to the website information. Further research is needed to study how exposure to health information on such websites influences patients' behavior.

### Conclusions

In summary, our study indicates that website information from both the American Society of Clinical Oncology and the Susan G Komen Breast Cancer Foundation appears to be appropriate, understandable, and accessible to multiethnic, multilingual cancer patients in public hospital settings. If issues related to finding such appropriate sites are addressed, these sites may represent a valuable resource for cancer information in such patient populations.

## Abbreviations

ASCO: American Society of Clinical Oncology
SKF: Susan G Komen Breast Cancer Foundation
